# Supplementary material for: Information Survey on the Use of Complementary and Alternative Medicine
Source: Medicina (Kaunas). 2022 Jan 14;58(1):125. doi: 10.3390/medicina58010125 (PMC8781067; doi:10.3390/medicina58010125)
Supplement: Supplementary file 1 [file medicina-58-00125-s001.zip › medicina-1448954-supplementary.pdf]

Supplementary Material

Supplementary Material Table S1. Summary of recommendations in which a consensus was reached for agreement.

**Recommendations**	**%**
CAMs can represent a useful supplement to conventional medical, surgical and rehabilitation therapies	97,4
Acupuncture must be performed by graduates in Medicine and Surgery, qualified to practice the profession of Doctor-Surgeon or Dentist	96,2
Homeopathy must be performed by graduates in Medicine and Surgery, qualified to practice the profession of Doctor-Surgeon or Dentist	96,2
Homotoxicology must be performed by graduates in Medicine and Surgery, qualified to practice the profession of Doctor-Surgeon or Dentist	96,2
Phytotherapy must be performed by graduates in Medicine and Surgery, qualified to practice the profession of Doctor-Surgeon or Dentist	80,8
Traditional Chinese Medicine must be performed by graduates in Medicine and Surgery, qualified to practice the profession of Doctor-Surgeon or Dentist	87,2
Ayurvedic Medicine must be performed by graduates in Medicine and Surgery, qualified to practice the profession of Doctor-Surgeon or Dentist	82,1
Medical diagnosis must always precede the use of CAMs	91,0
The use of CAMs does not conflict with the provisions of the Decree-Law of 07 June 2017, no. 73 "Urgent provisions on vaccine prevention", known as the vaccine decree	88,5
The integration between medical, surgical and rehabilitative therapies of Conventional Medicine and CAMs can be performed simultaneously in the same patient, if the clinical situation, and the medical indication makes it possible and necessary	94,9
The treatment of muscle-skeletal pathologies can benefit from the therapeutic integration of conventional medicine with acupuncture	93,6
The treatment of muscle-skeletal pathologies can benefit from the therapeutic integration of conventional medicine with homeopathy	84,6
The treatment of muscle-skeletal pathologies can benefit from the therapeutic integration of conventional medicine with homotoxicology	97,4
The treatment of muscle-skeletal pathologies can benefit from the therapeutic integration of conventional medicine with osteopathy	91,0
The treatment of muscle-skeletal pathologies can benefit from the therapeutic integration of conventional medicine with phytotherapy	75,6
The treatment of dermatological pathologies can benefit from the therapeutic integration of conventional medicine with homeopathy	88,5
The treatment of dermatological pathologies can benefit from the therapeutic integration of conventional medicine with homotoxicology	97,4
The treatment of dermatological pathologies can benefit from the therapeutic integration of conventional medicine with phytotherapy	82,1

The treatment of respiratory system pathologies can benefit from the therapeutic integration of conventional medicine with homeopathy	88,5
The treatment of respiratory pathologies can benefit from the therapeutic integration of conventional medicine with homotoxicology	94,9
The treatment of diseases of the respiratory system can benefit from the therapeutic integration of conventional medicine with phytotherapy	76,9
The treatment of allergic / immunological pathologies, even for the purpose of prevention, can benefit from the therapeutic integration of conventional medicine with homeopathy	94,9
The treatment of allergic / immunological pathologies, even for the purpose of prevention, can benefit from the therapeutic integration of conventional medicine with homotoxicology	97,4
The treatment of endocrine pathologies can benefit from the therapeutic integration of conventional medicine with homeopathy	82,1
The treatment of endocrine pathologies can benefit from the therapeutic integration of conventional medicine with homotoxicology	92,3
The treatment of headaches and neuralgia of the head and neck district can benefit from the therapeutic integration of conventional medicine with acupuncture	96,2
The treatment of headaches and neuralgia of the head and neck district can benefit from the therapeutic integration of conventional medicine with homeopathy	88,5
The treatment of headaches and neuralgia of the head and neck district can benefit from the therapeutic integration of conventional medicine with homotoxicology	96,2
The treatment of headaches and neuralgia of the head and neck district can benefit from the therapeutic integration of conventional medicine with osteopathy	92,3
The treatment of neuro-psychiatric pathologies can benefit from the therapeutic integration of conventional medicine with homeopathy	75,6
The treatment of neuro-psychiatric pathologies can benefit from the therapeutic integration of conventional medicine with homotoxicology	78,2
CAMs can be useful in the combined therapeutic approach of the patient with oncological pathology in order to contain the side and adverse effects of commonly used chemotherapy and / or radiotherapy therapies	84,0
CAMs can be useful in the combined therapeutic approach of the patient with oncological pathology in order to support the patient from a psychological point of view	81,3
CAMs MUST NOT be used exclusively in pathologies that require replacement therapy (eg Dependent Insulin Diabetes Mellitus)	89,7
In the therapy of nociceptive pain, the use of acupuncture in combination with conventional medical, surgical and rehabilitation therapies is advisable	96,2
In the treatment of nociceptive pain, the use of homotoxicology in combination with conventional medical, surgical and rehabilitation therapies is advisable	91,0
In the treatment of nociceptive pain, the use of osteopathy in combination with conventional medical, surgical and rehabilitation therapies is recommended	80,8
In the treatment of neuropathic pain, the use of acupuncture in combination with conventional medical, surgical and rehabilitation therapies is advisable	92,3

In the treatment of neuropathic pain, the use of homotoxicology in association with conventional medical, surgical and rehabilitation therapies is recommended	89,7
In the treatment of neuropathic pain, the use of osteopathy in association with conventional medical, surgical and rehabilitation therapies is advisable	75,6
In the treatment of muscle-skeletal pain, the use of acupuncture in combination with conventional medical, surgical and rehabilitation therapies is recommended	96,2
In the therapy of muscle-skeletal pain, the use of homeopathy in combination with conventional medical, surgical and rehabilitation therapies is recommended	85,9
In the therapy of muscle-skeletal pain, the use of homotoxicology in combination with conventional medical, surgical and rehabilitation therapies is recommended	94,9
In the therapy of muscle-skeletal pain, the use of osteopathy in combination with conventional medical, surgical and rehabilitation therapies is recommended	89,7
If the symptomatology of the patient treated exclusively with CAMs worsens persistently, it is correct not to continue with the treatment undertaken and to resort to conventional medical-surgical therapies.	88,5
In the case of pathologies affecting the musculoskeletal system, it is possible to use even more than one CAMs on the same patient with a combined approach	98,2 *
CAMs can be used in the head and neck district headache and neuralgia patient to facilitate the pharmacological "wash-out" in case of overlap of drug abuse headache	87,3 *
The use of Traditional Chinese Medicine as a therapeutic supplement to conventional medicine in the treatment of muscle-skeleton pathologies	87,3 *
The use of Traditional Chinese Medicine as a therapeutic supplement to conventional medicine in the treatment of dermatological pathologies	78,2 *
The use of Traditional Chinese Medicine as a therapeutic supplement to conventional medicine in the treatment of diseases of the respiratory system	85,5 *
The use of Traditional Chinese Medicine as a therapeutic supplement to conventional medicine in the treatment of allergic / immunological diseases	81,8 *
The use of Traditional Chinese Medicine as a therapeutic supplement to conventional medicine in the treatment of headaches and neuralgia of the head and neck district	89,1 *
The use of Traditional Chinese Medicine as a therapeutic supplement to conventional medicine in the treatment of gastroenteric diseases	85,5 *
The use of Traditional Chinese Medicine as a therapeutic supplement to conventional medicine in the treatment of nephro-urological diseases	76,4 *
The use of chiropractic as a therapeutic supplement to conventional medicine in the treatment of muscle-skeleton pathologies	85,5 *
The use of chiropractic as a therapeutic supplement to conventional medicine in the treatment of headaches and neuralgia of the head and neck district	81,8 *
The use of acupuncture as a therapeutic supplement to conventional medicine in the treatment of diseases of the respiratory system	78,2 *
The use of acupuncture as a therapeutic supplement to conventional medicine in the treatment of allergic / immunological diseases	76,4 *
The use of phytotherapy as a therapeutic supplement to conventional medicine in the treatment of allergic / immunological diseases	85,5 *

The use of phytotherapy as a therapeutic supplement to conventional medicine in the treatment of endocrine diseases	78,2 *
The use of phytotherapy as a therapeutic supplement to conventional medicine in the treatment of gastroenteric diseases	87,3 *
The use of phytotherapy as a therapeutic supplement to conventional medicine in the treatment of nephro-urological diseases	78,2 *
The use of non-conventional medicines are not frequent if you use acupuncture	83,6 *

CAM: Complementary and alternative medicine; *Recommendations derived from Q2.

Supplementary Material Table S2. Summary of recommendations in which a consensus was reached for disagreement.

**Recommendations**	%
Complications due to the use of CAMs are frequent if homeopathy is used	84,6
Complications due to the use of CAMs are frequent if homotoxicology is used	83,3

CAM: Complementary and alternative medicine
